# Epigenetic differences of chronic hepatitis B in different TCM syndromes: Protocol for a case-control, non-interventional, observational clinical study

**DOI:** 10.1097/MD.0000000000012452

**Published:** 2018-09-28

**Authors:** Li Ma, Xiuli Zheng, Yu Yang, Jian Wang, Youli Xu, Baojia Wang

**Affiliations:** a Chengdu University of Traditional Chinese Medicine, Chengdu, Sichuan; b Ningxia Medical University, Yinchuan, Ningxia; c Pixian Hospital of Traditional Chinese Medicine, Chengdu, Sichuan, China.

**Keywords:** chronic hepatitis B, DNA methylation, epigenetics, TCM syndromes

## Abstract

**Introduction:**

Chronic hepatitis B is a serious disease causing serious harm to the human health. Chinese medicine has its unique advantages in the clinical prevention and treatment, while the syndrome of Chinese medicine lacks the understanding at the micro level. There are some theoretical commonalities between the epigenetics and traditional Chinese medicine (TCM) syndromes. The biological basis of chronic hepatitis B (CHB) syndrome differentiation from the perspective of epigenetics is of great significance to diagnose and prevent the diseases.

**Methods:**

This protocol is a case-control, noninterventional, observational clinical study. Patients with CHB for spleen-stomach damp heat and liver depression and spleen deficiency, with 12 each and 11 healthy volunteers were recruited. Peripheral venous blood was collected from the participants. DNA methylated transferase, genomic DNA methylated spectrum, methylated DNA binding protein MeCP2, chronic infection of hepatitis B virus with methylated related proteins, and miRNA target genes were analyzed.

**Objectives:**

From the perspective of DNA methylation epigenetics, “DNA methylation–miRNA–Target gene” is the main line, which further reveals the essence of TCM syndrome. To improve the level of TCM clinical syndrome differentiation and the clinical efficacy of TCM, especially in the study of TCM syndromes of CHB, discovering its underlying biological signature is necessary.

**Trial registration:**

Clinical Trials Registration: ChiCTR1800017365, registered 26 July 2018.

## 1 Introduction

Chronic hepatitis B (CHB) is an infectious disease caused by hepatitis B virus (HBV), and the liver disease caused by infection is the most common type of chronic hepatitis, posing a serious threat to human health. According to a recent study report, 3.6% of the world's population is chronically infected with HBV,^[1]^ and 73% of liver cancer deaths are provoked by HBV and hepatitis C virus infections.^[2]^ There are more than 120 million HBV carriers in China, and nearly 300,000 HBV carriers die each year from cirrhosis and liver cancer.^[3]^ Traditional Chinese medicine (TCM) has long been used in the clinical prevention and treatment of CHB. However, to further improve the ability of TCM in the prevention and treatment of CHB, it is necessary to break the bottleneck of “interpretation of the basis of syndrome biology.”

The TCM syndrome is a pathological generalization of the clinical symptoms and signs of a disease, at a certain stage, under the guidance of TCM theory. Syndrome is characterized by facultative, dynamic, and complexity of symptoms, and lack microcosmic level of understanding and is a dialectical method for the syndrome of objective (ie, syndrome formation and evolution mechanism and standard syndromes) challenges.^[4]^ So, research of CHB biology syndromes reveals the biological basis of different CHB syndromes and their characteristics. This, in turn, promotes the development of Chinese medicine, giving full play to the advantages of characteristics of TCM, further improving the ability of TCM in the prevention and treatment of CHB remains of great significance.

While the previous studies conducted on the mechanism of miRNA in CHB syndrome differentiation have achieved encouraging results, and the research group revealed that the same miRNA exists in different syndromes, which is difficult to explain. Studies have shown that miRNA and DNA methylation interact to regulate gene expression, and miRNA induced by DNA methylation can target MeCP2 and DNMTs directly or indirectly.^[5]^ Therefore, it is speculated that the reason for different types of syndromes in the same miRNA may be related to the epigenetic regulation of DNA methylation. The factors associated for the formation of CHB include HBV gene polymorphism, immune-related gene polymorphism, and dysfunction of host immune cells.^[6]^ According to a recent study, both host and viral genome DNA methylation can influence HBV replication, the infected host cell epigenetic characteristics can be used as a kind of epigenetic markers, and the results can be predicted more accurately in diagnosing the infection or disease.^[7]^ DNA methylation and histone modification can regulate HBV gene expression and virus replication.^[8]^ HBV can be integrated into the host genome or form covalently closed circular DNA (cccDNA) microstaining. The persistent overexpression of cccDNA pools in HBV is related to DNA methyltransferase.^[9]^ Also, DNA methylation is specific to the CpG sites,^[10]^ where at least 6 CpG islands are present in the HBV genome.^[11]^ The CpG islands differ in their length and characteristics at different clinical stages of viral infection. Therefore, we proposed that differences in the DNA methylation level may be 1 of the main epigenetic mechanisms of CHB syndrome difference. Hence, this study further revealed the essence of TCM syndromes from the perspective of DNA methylation epigenetics to improve the level of TCM clinical syndrome differentiation and the clinical efficacy of TCM, especially in studying TCM syndromes of CHB, and discover its potential biological markers.

## 2 Methods

### 2.1 Ethics approval

The study protocol was approved by the Sichuan Regional Ethics Review Committee of TCM and Medical Ethics Committee of Affiliated Hospital of Chengdu University of TCM (No: 2018KL-057). This protocol has been registered in China clinical trial registry (No: ChiCTR1800017365). This protocol is in compliance with the declaration of Helsinki. All participants signed informed consent form before participation in the test. All participants’ personal information was collected, shared, and maintained in a separate space to protect confidentiality before, during, and after the trial.

### 2.2 Participants

Patients were recruited from the outpatient and inpatient departments of Pixian Hospital of TCM in Chengdu, Sichuan Province, and healthy volunteers were recruited from Chengdu, Sichuan Province.

#### 2.2.1 Diagnostic criteria

According to the diagnostic criteria of China CHB Prevention and Control Guide issued by the China Hepatitis Prevention Foundation, the diseases were classified into liver disease of the Chinese Medical Association and infectious diseases in October, 2015.

According to the TCM syndrome differentiation standard for viral hepatitis (trial) (1991), it is defined under the following:

1. Spleen-stomach damp heat syndrome: rib-side distention and pain; abdominal distention and sloppy stool; despondency, vexation, and oppression; fatigue and lack of strength; pale, teeth-marked tongue
2. Liver depression and spleen deficiency syndrome: stifling sensation in the epigastrium and abdomen; slimy yellow tongue fur; nausea, anaerobic oil, poor appetite, and digestion; yellowing of the body, eyes, and urine; sticky and dirty stools.

#### 2.2.2 Inclusion criteria

The inclusion criteria are as follows:

1. The patient should meet the diagnostic criteria of CHB and TCM syndrome differentiation criteria
2. Male or female, aged 18 to 60 years
3. The person who agrees and signs the informed consent

#### 2.2.3 Exclusion criteria

The exclusion criteria are as follows:

1. Concomitant with other types of hepatitis virus or/and HIV infection
2. Patients with cirrhosis and malignant tumors
3. Combination of drug-induced or toxic liver damage, autoimmune hepatitis, and heritable-metabolic liver diseases
4. Unclear consciousness, unable to express subjective discomfort symptoms and psychotic patients
5. Patients with serious diseases related to heart, lung, kidney, endocrine, and blood
6. If the researcher believes any other situations that are not suitable for the study

### 2.3 Trial design

This is a case-control, noninterventional, observational study. The study protocol was conducted in strict accordance with the guidelines for clinical trial protocol specification (SPIRIT2013). This protocol was supported by a project funded by the national natural science foundation of China (No: 81603515).

**Figure 1:**
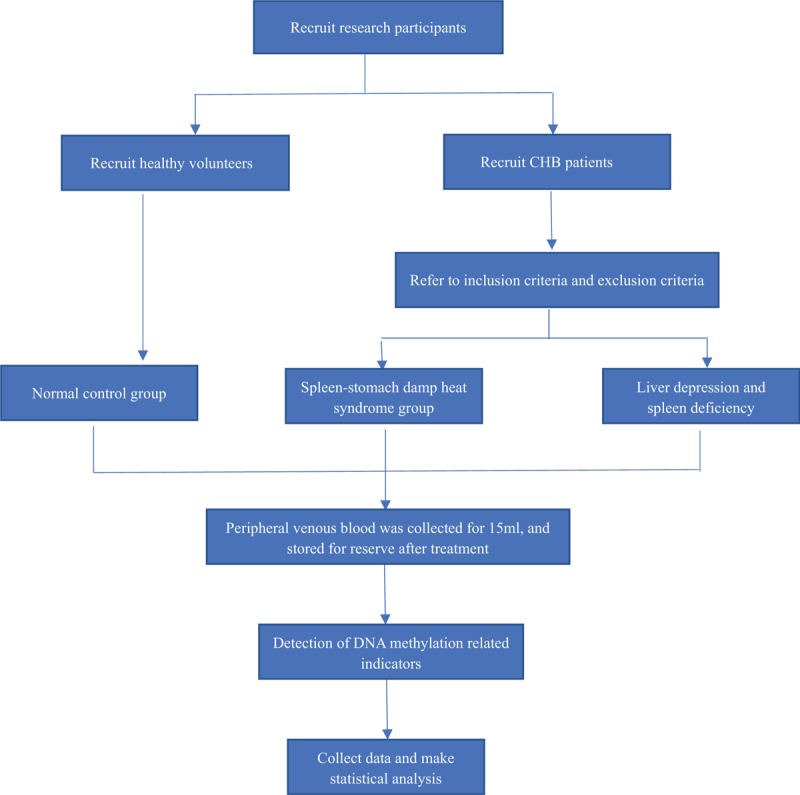
Study design flow chart. A chart shows the entire process of this clinical study.

This study recruited patients according to the CHB diagnostic standards, and after the dialectical treatment of TCM of spleen-stomach damp heat syndrome and liver depression and spleen deficiency syndrome, the participants were divided into CHB spleen-stomach damp heat syndrome group and liver depression and spleen deficiency syndrome group, with 12 cases in each group. Eleven healthy volunteers were recruited as controls. Participants had to sign informed consent form before participation in the study. In the fasting state, that is, before breakfast, 15 mL of peripheral venous blood was collected from the subjects. The overall test process is shown in Fig. 1.

### 2.4 Interventions

There were no interventions in this study.

### 2.5 Outcome measures

#### 2.5.1 Clinical data

All the samples were coded and only the researchers knew about the participants. Clinical information including general information on participants, laboratory tests, and disease history are recorded.

#### 2.5.2 Comparative analysis of DNA methylated transferase activity/inhibition in different syndromes

Blood samples of CHB patients were collected from the spleen-stomach damp heat syndrome group and liver depression and spleen deficiency syndrome group to analyze serum DNA methyltransferase (DNMT, including DNMT1, DNMT3A, and DNMT3B) activity, and provide reference for subsequent chip, high, and low methylation status results.

#### 2.5.3 Comparative analysis of DNA methylation spectra of different syndromes

The whole genome DNA extraction of the specimens, the application of genome-wide area promoter CpG island methylation chip research of the two syndromes of CHB patients with peripheral blood DNA methylation status compared DNA methylation status differences of the two syndromes.

#### 2.5.4 Comparative analysis of MeCP2 of different syndromes

MeCP2 is an important binding protein for DNA methylation. The content of MeCP2 in serum samples of patients with the 2 syndromes was detected, and compared the differences of MeCP2 in the 2 syndromes.

#### 2.5.5 Comparison of methylated related proteins in chronic HBV infection with different syndromes

The serum levels of hepatitis b virus X protein (HBx), E-cadherin (E-cad), p16 protein, and p53 protein in the 2 groups of symptomatic patients of the 2 syndromes were compared for the types of methylation-related proteins.

#### 2.5.6 Correlation analysis between DNA methylation levels of different syndromes and miRNAs target genes

The early miRNA research results from the liver of miR-122 specific miRNAs to observe the target genes of miR-122 cyclin G1expression in the 2 syndromes were combined with the DNA methylation level correlation analysis of the 2 syndromes to observe the same target genes of miRNAs in different syndromes under the influence of DNA methylation levels in the expression.

This was based on the following: miR-122 is a hepatic-specific miRNA^[12]^ and miR-122 can regulate HBV replication through its effect on target cell cycle protein Gl (cyclin G1). Some studies have shown that cyclinG1 affects on p53 protein, hindering the function of p53 binding HBV enhancer to inhibit HBV. Overexpression of miR-122 can eliminate the blocking effect of cyclinG1 on p53 protein and indirectly inhibit HBV replication.^[13]^

#### 2.5.7 Data fusion analysis of DNA methylation–miRNA–target genes

Firstly, the different syndromes of CHB patients, DNA methylation transferase, DNA methylation, DNA binding protein, and methylation of chronic HBV infection related protein data, comprehensive analysis of the methylation level differences between the patients with syndromes were observed. Secondly, according to the previous research results, 4 miRNAs were repeated in CHB syndrome of spleen-stomach damp heat and syndrome of liver depression and spleen deficiency, and the 2 syndrome types of DNA methylation level correlation analysis was performed to observe the same miRNAs in different syndrome types of DNA methylation level differences. Thirdly, the methylation level data and miR-122 target gene cyclin G1 data were combined for correlation analysis between the methylation levels of different syndromes of DNA and miRNAs target genes. This allowed full use of information from the public database, bioinformatics, mathematics, and other methods to conduct function mining, deepening the understanding of the epigenetic mechanism of different syndromes of CHB.

### 2.6 Sample size

The minimum sample number calculated in order to achieve the goals of the project was fully accomplished. Considering that about 90% of CHB diagnosis and TCM syndromes can be accurately classified, 5% error rate and 95% confidence interval (CI) were initially predicted for the sample size of 30 subjects. However, we have also considered a 15% rate of sample loss (defective sample), which subsequently generated a total of 35 participants for the recruitment.

### 2.7 Statistical analysis

Statistical analyses were performed using the SPSS statistical package program, version19.0. Each group adopted *t* test and chi-square test, and variables with *P* values less than .05 were considered to be statistically significant. The Carma method of clementine 12.0 was used to analyze the association rules between the groups.

### 2.8 Monitoring of the study

A qualified doctor monitored this study. The doctor ensured that all the procedures were carried out, recorded, and reported in accordance with the standard operating procedures and all applicable regulatory requirements. Because this is a noninterventional study, it represents no risk or benefit for the patient.

## 3 Discussion

Epigenetics is a hot research topic in recent years, which mainly includes histone modification, noncoding RNA, and DNA methylation. DNA methylation is the most common and the most studied epigenetic content.^[14]^ DNA methylation is a chemical modification process where the methyltransferases (DNMTs) are catalyzed by selective addition of methyl groups to form 5-methylcytosine in CpG sequences.^[10]^ Studies^[7–11]^ have shown the association of DNA methylation to the occurrence and development of CHB.

**Figure 2:**
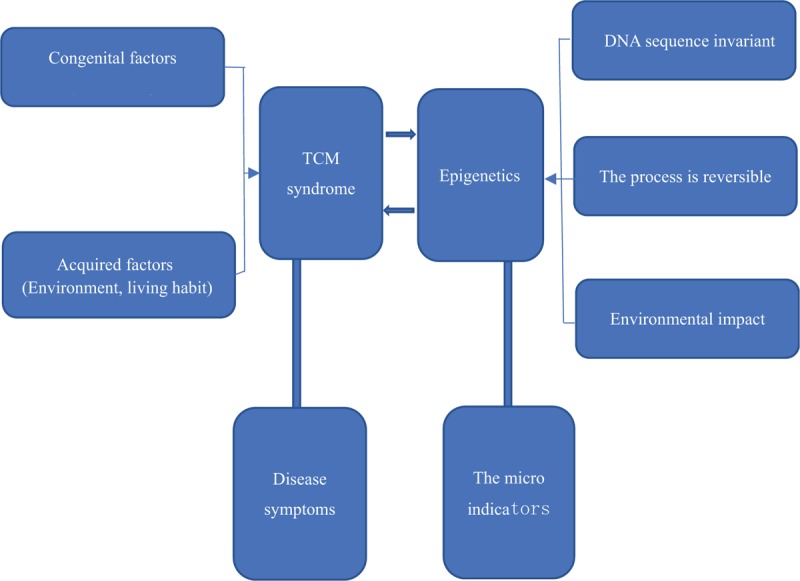
Correlation between TCM syndromes and epigenetics. Epigenetics is the partial material basis of TCM syndrome diversity, and the microscopic index of epigenetics can be a necessary supplement to the macroscopic syndrome differentiation of TCM syndromes. TCM = traditional Chinese medicine.

Epigenetics refers to the heritable changes that do not change the underlying DNA sequences, but the gene expression and the functions change, and the process is reversible and environmental.^[15]^ TCM holds that syndromes are the generalization of the pathological characteristics of human body at various stages under the influence of internal and external environment, which is similar with the “microscopic concept” of epigenetics. Some scholars have proposed that epigenetics is the partial material basis of TCM syndrome diversity, and the microscopic index of epigenetics can be a necessary supplement to the macroscopic syndrome differentiation of TCM syndromes^[16]^ (Fig. 2). Therefore, it is considered as one of the key scientific problems to realize the breakthrough of TCM clinical practice and the theory to elucidate the molecular biological basis of TCM syndromes from the correlation between epigenetics and TCM syndromes phenotypes.^[17]^

In recent years, preliminary progress has been made in exploring the essence of TCM syndrome from the perspective of DNA methylation. For example, in patients with acute leukemia, which compared with the dual vacuity of Qi and Yin syndrome, ID4 gene methylation is more likely to occur in patients with toxic hot flaming syndrome and static blood and binding phlegm syndrome.^[18]^ Differences in methylation of HLA-DRB1 and ADAMTS9, NUDT1 and YES1, and APOA5 and PRKCZ genes might be related to hepatitis B and cirrhosis with damp-heat brewing internally syndrome, spleen deficiency, and exuberant phlegm and liver-kidney yin depletion.^[19]^ The methylation of the promoter regions of FHIT, MAP2K6, WNT5B, CSNK1D, and FRAT2 genes on CpG island was associated with kidney yang vacuity.^[20]^ At present, the research of molecular mechanism of DNA methylation is still at the initial stage. The biological basis of syndrome is largely confined to a single or a few genes and the relationship between syndrome and lack of whole genome methylation level to interpret the essence of the same disease syndrome is still under research. The research team studied the distribution characteristics of CHB syndrome types in Sichuan China, and found that the syndrome of spleen-stomach damp heat and liver depression and spleen deficiency are the most common syndromes of CHB in TCM syndrome.^[21]^ Therefore, this study has chosen “DNA methylation–miRNA–target genes” as the main line of research. For the first time (not reported), our study revealed from the perspective of DNA methylation epigenetics of CHB spleen-stomach damp heat and liver depression and spleen deficiency syndrome differences between biological basis, providing a preliminary basis for the classification of CHB TCM syndromes to promote its objectification and standardization. This, in turn, provides a theoretical basis for the diagnosis, prevention, and treatment of diseases and the development of new drugs. At the same time, the study has fewer sample size as limitation, and the DNA methylation-specific differences between different syndromes require large sample validation during the follow-up. Also, DNA methylation in specific mechanisms of formation and development of syndromes still need further discussion.

## 4 Consent for publication

All participants in the study have signed a consent form for publication of data.

### 4.1 Availability of data and material

The datasets used and/or analyzed during the current study are available from the corresponding author on reasonable request.

## Acknowledgment

The authors thank the participating patients of the study, and the University Hospital medical and nursing staff.

## Author contributions

X.L. Zheng was designed the research. Y. Yang was guided the whole research. L. Ma and X.L. Zheng designed the study protocol and wrote the manuscript. X.L. Zheng and L. Ma sought financial and ethical approval. J.Wang, L. Ma and L.Y. Xu were collected from standing cases and specimens. Y. Yang, X.L. Zheng and L. Ma reviewed and revised the manuscript. B.J. Wang was responsible for statistical analysis. All authors read and approved the final manuscript. The corresponding author is only responsible for submitting the published manuscript.

**Conceptualization:** Li Ma, Xiuli Zheng.

**Data curation:** Youli Xu.

**Formal analysis:** Xiuli Zheng, Baojia Wang.

**Funding acquisition:** Li Ma, Xiuli Zheng.

**Investigation:** Li Ma, Xiuli Zheng, jiang wang.

**Methodology:** Li Ma, Xiuli Zheng.

**Project administration:** Yu Yang.

**Supervision:** Yu Yang.

**Visualization:** Li Ma, Xiuli Zheng.

**Writing – original draft:** Li Ma.

**Writing – review & editing:** Xiuli Zheng, Yu Yang.
